# Partitioning of Antibiotic Resistance Genes and Fecal Indicators Varies Intra and Inter-Storm during Combined Sewer Overflows

**DOI:** 10.3389/fmicb.2017.02024

**Published:** 2017-10-20

**Authors:** Alessia Eramo, Hannah Delos Reyes, Nicole L. Fahrenfeld

**Affiliations:** Department of Civil and Environmental Engineering, Rutgers, The State University of New Jersey, Piscataway, NJ, United States

**Keywords:** settleable particles, hydrodynamic separation, stormwater, ARG, amplicon sequencing, wastewater biomarker, CSO

## Abstract

Combined sewer overflows (CSOs) degrade water quality through the release of microbial contaminants in CSO effluent. Improved understanding of the partitioning of microbial contaminants onto settleable particles can provide insight into their fate in end-of-pipe treatment systems or following release during CSO events. Sampling was performed across the hydrograph for three storm events as well as during baseflow and wet weather in three surface waters impacted by CSO. qPCR was performed for select antibiotic resistance genes (ARG) and a marker gene for human fecal indicator organisms (BacHum) in samples processed the partitioning of microbial contaminants on settleable particles versus suspended in the aqueous phase. Amplicon sequencing was performed on both fractions of storm samples to further define the timing and partitioning of microbial contaminants released during CSO events. Samples collected at the CSO outfall exhibited microbial community signatures of wastewater at select time points early or late in the storm events. CSOs were found to be a source of ARG. In surrounding surface waters, *sul*1 was higher in samples from select locations during wet weather compared to baseflow. Otherwise, ARG concentrations were variable with no differences between baseflow and wet weather conditions. The majority of ARG at the CSO outfall were observed on the attached fraction of samples: 64–79% of *sul*1 and 59–88% of *tet*(G). However, the timing of peak ARG and human fecal indicator marker gene BacHum did not necessarily coincide with observation of the microbial signature of wastewater in CSO effluent. Therefore, unit processes that remove settleable particles (e.g., hydrodynamic separators) operated throughout a CSO event would achieve up to (0.5–0.9)-log removal of ARG and fecal indicators by removing the attached fraction of measured genes. Secondary treatment would be required if greater removal of these targets is needed.

## Introduction

Outdated sewer infrastructure results in the release of 26 × 10^6^ m^3^ of untreated wastewater to New Jersey (NJ) surface waters each year (New Jersey Future, 2017). Combined sewer overflows (CSOs) degrade water quality and present a risk to public health because they discharge priority pollutants (Gasperi et al., 2008), nutrients (Gervin and Brix, 2001), pathogens, and fecal bacteria (Donovan et al., 2008) into waterways. Incidental ingestion of CSO-plagued Passaic River water was associated with an elevated risk of gastrointestinal disease (Donovan et al., 2008). Solutions are needed to remove or inactivate a broad suite of microbial contaminants in CSO effluent.

End-of-pipe treatment of CSO effluent is a potential solution for improving water quality at a lower cost than upgrading combined sewer systems to separate sanitary systems, which is estimated to cost $40.8 billion in the United States (United States Environmental Protection Agency, 2016). End-of-pipe treatment systems for CSO effluent range from screening for floatables to more advanced unit processes such as rapid filtration and disinfection. Hydrodynamic or vortex separators are end-of-pipe treatment technologies that target the removal of settleable particles primarily through extension of the particle flow path, allowing a longer time for gravitational forces to remove settleable particles (Alkhaddar et al., 2001; Andoh and Saul, 2003). Therefore, understanding the timing and partitioning of microbial contaminants on settleable particles in CSO effluent on settleable particles is needed for designing and operating end-of-pipe treatment systems. Quantifying the partitioning of microbial contaminants can also aid in determining the fate of microbial contaminants released to surface waters during storm events given that microbes attached to settleable particles will have different fates than those on non-settleable particles and planktonic cells (Krometis et al., 2007). For CSO (Passerat et al., 2011) and storm events (Krometis et al., 2007), intra-storm variability in the proportion of attached and suspended microbes has been demonstrated for a limited set of indicator organisms. Understanding the timing and potential for removal of indicator organisms is pertinent for meeting water quality regulations of the Clean Water Act. However, because correlations between indicator organisms and pathogens are often weak (Harwood et al., 2005) and different microbes associate with particles at different rates (Suter et al., 2011), targeting a broader range of microbial contaminants is of interest.

Antibiotic resistance genes (ARG) are emerging microbial contaminants of concern in the environment given that environmental sources of antibiotic resistance have been linked to clinical infections (Forsberg et al., 2012). High levels of antibiotic resistant bacteria in the CSO-impacted Hudson River were associated with wet weather (Young et al., 2013). Determining the relative importance of different sources of antibiotic resistance in our waterways is key for developing mitigation strategies. Yet, little is known about the concentration, timing, and partitioning of antibiotic resistant bacteria and ARG in CSO (McLellan et al., 2007; Fahrenfeld and Bisceglia, 2016), limiting our understanding of the importance of CSO as a source of these contaminants and our ability to evaluate end-of-pipe treatment technologies. The association of ARG with colloidal material has been documented in wastewater (Breazeal et al., 2013) and elevated levels of ARG and antibiotic resistant bacteria have been observed in biofilm in various water environments (Zhang et al., 2009), indicating that the partitioning of ARG onto settleable particles in CSO may be an important control on their fate.

The aim of this work is to expand our understanding of the intra- and inter-storm variability in the partitioning of a broader suite of microbial contaminants onto settleable particles during CSO events. To achieve this goal, water samples were collected across three CSO events and settleable and suspended bacteria were separated using previously validated methods (Characklis et al., 2005; Krometis et al., 2007). Analysis was performed for a human fecal indicator marker gene (BacHum) using qPCR to determine the timing of the release of fecal microbes during the CSO events and to allow for comparison to other studies of the partitioning of fecal indicators during CSO and storm events. Amplicon sequencing was performed to broaden our understanding of the variation of microbial communities released throughout a CSO event. To understand the role of CSO as a source of ARG, select ARG found in wastewater encoding for sulfonamide and tetracycline resistance were analyzed using qPCR during the CSO events and compared to those observed in local waterways in different flow conditions (i.e., baseflow and wet weather). Finally, the intra- and inter-storm variability of the partitioning of ARG observed in the CSO was quantified to provide insight into the potential for treatment processes that remove settleable particles to remove these emerging contaminants.

## Materials and Methods

### Sampling and Water Quality Analysis

**FIGURE 1:**
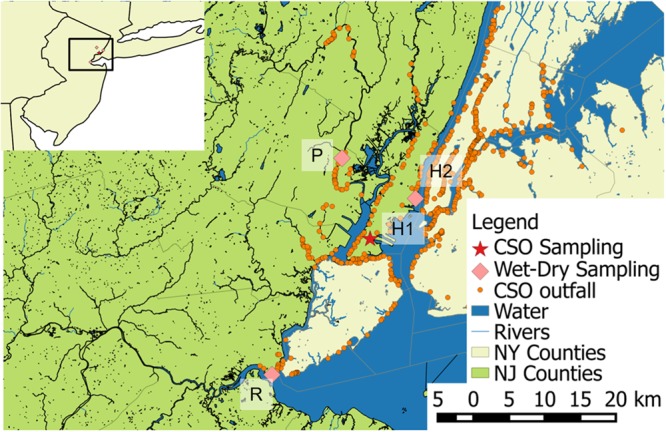
Map of sampling locations for wet weather sampling in three waterways impacted by combined sewer overflows (CSOs), location of CSO outfall sampled across precipitation events, and documented CSO outfalls. R is on the Raritan River, P on the Passaic River, and H1 and H2 on the Hudson River and Bay.

CSO effluent was collected during three storm events in the surface water outside of a CSO outfall in northern NJ (**Figure 1** and Supplementary Figure A1). The outfall sampled has a drainage area that is 98% urban and 57% impervious. The outfall had 78 discharges in 2014 (Van Abs, 2014) and ∼0.5 cm of rainfall was sufficient to cause CSO at another outfall in this system during the months when sampling took place. During Storm I (2.4 cm, 8/11/15), duplicate samples (1 L) were collected at two time points. For Storm II (2.5 cm, 9/10/15) and Storm III (3.6 cm, 10/28/15), singlet samples (1 L) were collected every 15–30 min, based on the forecasted duration of the storm. The high similarity of the Storm I duplicate samples motivated singlet sampling to allow for greater temporal coverage during Storms II and III. All samples for biomolecular analysis were stored on ice until processing in the laboratory. Conductivity and pH were measured in the field with a multimeter (Orion Star A329, Thermo Scientific). Aliquots (20 mL) of samples from each time point were analyzed for total suspended solids (TSS), using Environmental Sciences Section Method 340.2 (Wisconsin State Lab of Hygiene, 1993). Field blanks consisting of autoclaved deionized water left open for the duration of Storms II and III sampling and otherwise treated as field samples were analyzed for QA/QC. Historical rainfall data was collected from Weather Underground (The Weather Company, 2017).

To separate settleable and suspended microbes, methods previously validated by Krometis et al. (2007) were applied. The two Storm I and seven Storm II samples were analyzed directly. The 10 field samples from Storm III were composited in the lab to generate five representative samples from across the precipitation event. Briefly, samples (∼0.7 to 1 L) were centrifuged at 1160 × *g* for 10 min at 4°C. The supernatant (top 70% volume fraction) was collected by pipette and will be referred to as “suspended.” The suspended fraction was previously determined to contain 80% of non-settleable particles in stormwater (Characklis et al., 2005). The remaining fraction represents the settleable particles and will be referred to as “attached.” After separating these two fractions, each sample was filter concentrated (0.22 μm, nitrocellulose) and stored at -20°C prior to DNA extraction. To determine the microbial community structure indicative of untreated wastewater, post-screening grab samples of wastewater influent were collected (10/26/16) from a municipal wastewater treatment plant (WWTP) outside of the studied sewer systems, diluted with sterile DI water (23% wastewater to simulate CSO effluent), and either pelleted by centrifugation at 4000 × *g* for 15 min or filter concentrated (0.22 μm, nitrocellulose).

To compare the concentration of ARG in CSO effluent to that in local waterways under different flow and tidal conditions, water (1 L) and composited bed sediment (∼50 mL) samples were collected in triplicate during baseflow (April 6 or 16, 2015) and during or following wet weather (June 1, 2015, Supplementary Figure A2) in three surface waters (Hudson River, Raritan Bay, and Passaic River) (**Figure 1**). The mean daily temperature for baseflow and wet weather sampling events was ± 2°C. Nearby rainfall gauges reported cumulative rainfall of 1.8 cm for wet weather sampling in the Passaic River and Raritan Bay and 4.2 cm for the wet weather sampling on the Hudson River, all of which were sufficient to cause CSO. The Passaic and Hudson Rivers are tidally influenced at the sampling locations. Both the baseflow and wet weather sampling occurred during low tide in the Passaic River. The Hudson River baseflow sampling occurred during high tide, while the wet weather sampling occurred during low tide. The Raritan Bay baseflow sampling occurred during low tide and wet weather sampling occurred during high tide. Aqueous samples (∼500 to 900 mL) were concentrated on 0.22 μm nitrocellulose filters (Millipore Corporation, Billerica, MA, United States) prior to DNA extraction. Sediment samples were homogenized prior to DNA extraction, and analyzed as described in Section “Biomolecular Analyses.”

### Biomolecular Analyses

DNA was extracted from filter concentrated samples (wastewater, water from baseflow and wet weather sampling, and the suspended and attached fractions of storm samples), cell pellets (0.5 mL of cell pellet from wastewater samples), or sediment (∼0.5 g) using a commercial kit (FastDNA Spin Kit for Soil, MP Biomedicals) following the manufacturer’s directions. To determine the concentration of ARG across the precipitation event, qPCR was performed on Storms I-III samples for select ARG [*sul*1, *tet*(G)], BacHum as a human fecal indicator (Kildare et al., 2007) and 16S rRNA gene copies for total bacterial population (Zhang et al., 2016). Baseflow and wet weather samples were subjected to qPCR analysis for select ARG [*sul*1 and *sul*2 (Pei et al., 2006), *tet*(G) and *tet*(O) (Aminov et al., 2002)] and 16S rRNA gene copies as a surrogate for total bacterial population (Suzuki et al., 2000). A standard SYBR Green (5 μL SsoFast EvaGreen, Bio-Rad, Hercules, CA, United States) chemistry with 0.4 μM forward and reverse primers, and 1 μL diluted (1:100) DNA extract in a 10 μL reaction was used for all genes except BacHum. Probe chemistry (5 μL SsoAdvanced Universal Probes Supermix, Bio-Rad, Hercules, CA, United States) with 0.22 μM of each primer, 0.07 μM of probe, and 1 μL diluted (1:100) DNA extract was used for BacHum in a 10 μL reaction. QA/QC performed during qPCR included analyzing a no-template control on each plate, a seven point calibration curve, and melt curve and/or gel electrophoresis to verify the specificity of qPCR products. Amplicon sequencing (Illumina MiSeq, 300 bp, paired end) was performed on samples of WWTP influent and from the three storms targeting the V3-4 region of the 16S rRNA gene at a commercial lab (MR DNA, Shallowater, TX, United States). Sequences were analyzed using mothur MiSeq Standard Operating Procedure (Accessed 9/2016) (Kozich et al., 2013).

### Statistical Analyses

To test for differences in wet weather versus baseflow conditions, a Wilcoxon rank sum test was applied to ARG copies normalized to the 16S rRNA gene copies. A paired Student’s *t*-test (normality of data confirmed by a Shapiro test) or a Wilcoxon rank sum test was applied to test for differences in attached and suspended log-normalized ARG or 16S rRNA gene copy numbers intra-storm. A Kruskal–Wallis rank sum test followed by a *post hoc* pairwise *t*-test was performed on log-normalized total gene copy numbers to test for inter-storm differences. These statistical tests were performed in R (R Core Team, 2013). A Bray–Curtis similarity matrix was calculated on log-normalized subsampled (*N* = 14,052 sequences) operational taxonomic unit data at the class level followed by cluster analysis with a SIMPROF test and non-metric multidimensional scaling (nMDS) in PRIMER 7. Rarefaction was performed in mothur. To determine which taxa were preferentially associated with a given sample type (settleable storm, suspended storm, wastewater) biomarker analysis was performed on class-level relative abundance data for samples excluding the storm samples that formed significant clusters with wastewater. The linear discriminant analysis effect size (LEfSe) tool (Segata et al., 2011) was used to identify biomarkers using the default settings.

## Results

### Partitioning of ARG and Microbial Contaminants across the Hydrograph

**FIGURE 2:**
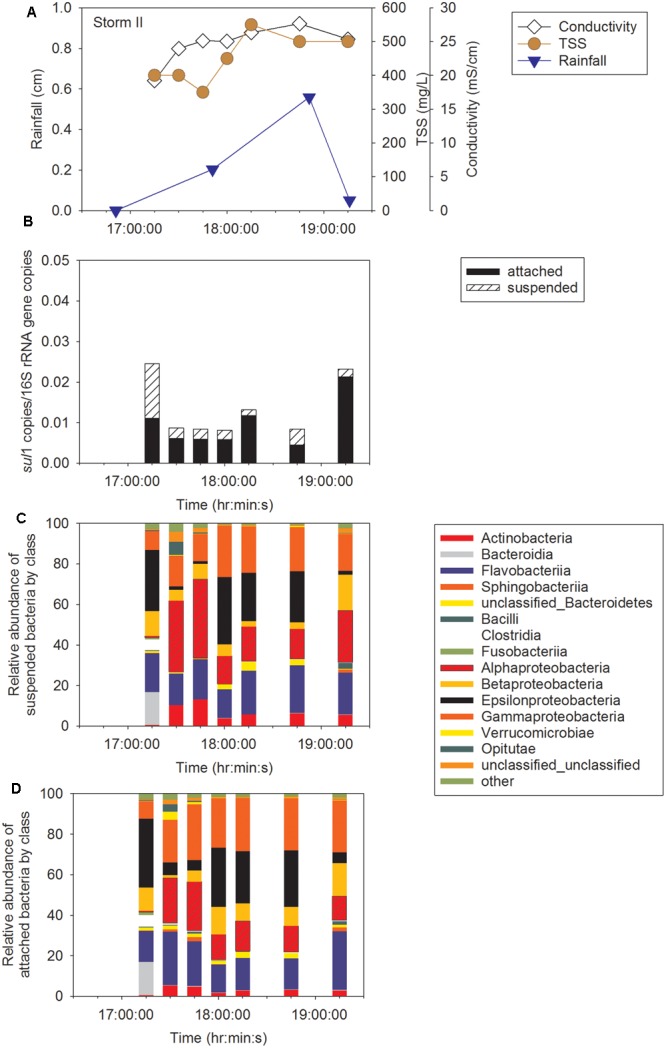
Storm II **(A)** rainfall, total suspended solids (TSS), and conductivity, **(B)** *sul*1, **(C)** suspended bacteria by class, and **(D)** attached bacteria by class.

**FIGURE 3:**
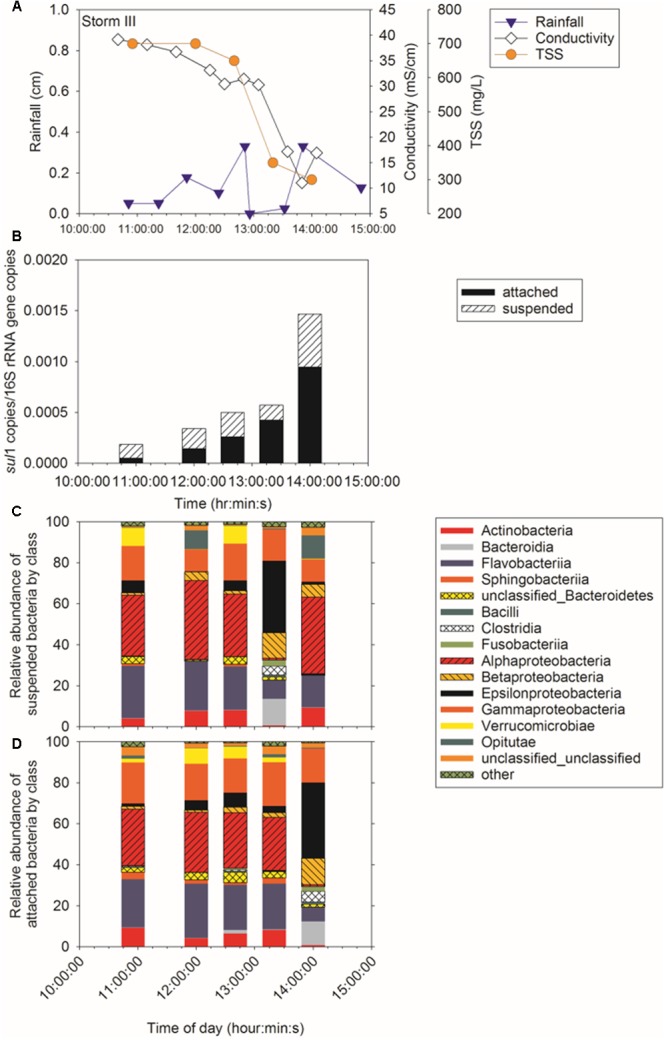
Storm III **(A)** rainfall, TSS, and conductivity, **(B)** *sul*1, **(C)** suspended bacteria by class, and **(D)** attached bacteria by class.

**FIGURE 4:**
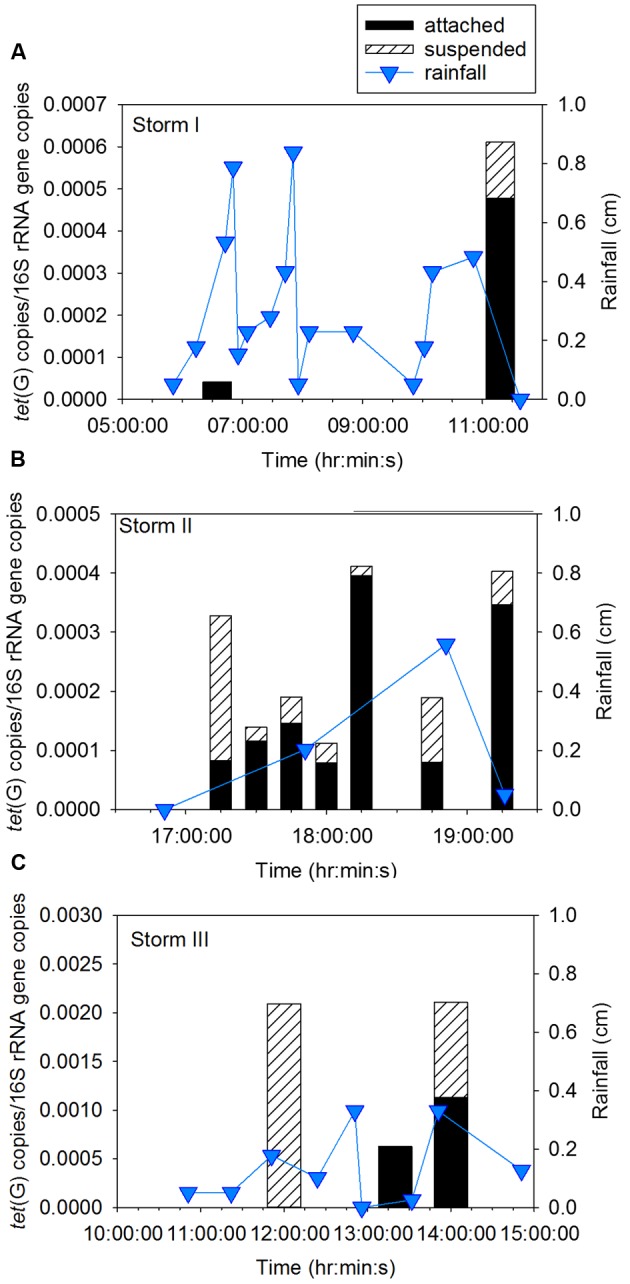
Attached and suspended *tet*(G) gene copies/16S rRNA gene copies and rainfall across **(A)** Storm I, **(B)** Storm II, and **(C)** Storm III. Storm I results represent the average of duplicate samples with a relative percent difference of ±1.6 to 20.5%.

Sampling during three storm events was performed to determine concentration of ARG in CSO effluent, the proportion of attached compared to suspended ARG, and the intra- and inter-storm variability in this partitioning. Attached *sul*1 gene copies accounted for 64% ± 6% of the total *sul*1 gene copies for Storm I (Supplementary Figure A3), 79% ± 6% of the total *sul*1 gene copies for Storm II (**Figure 2**), and 71% ± 18% of the total *sul*1 gene copies for Storm III (**Figure 3**). Across all storms, attached *sul*1 gene copies were greater than suspended *sul*1 gene copy concentrations (*p* = 1.1 × 10^-5^). For *tet*(G), attached gene copy concentrations were greater than suspended for the three storms (*p* = 0.03) with the attached fraction accounting for 88% ± 9% of total *tet*(G) gene copies during Storm I, 77% ± 13% of total *tet*(G) gene copies for Storm II, and 59% ± 52% of total *tet*(G) gene copies for Storm III (**Figure 4**). *tet*(G) was not detected in all Storm III samples or in both phases when it was detected. Normalizing *tet*(G) concentrations to 16S rRNA gene copy numbers resulted in no difference in the partitioning between attached and suspended phases across the storms (*p* = 0.15).

**FIGURE 5:**
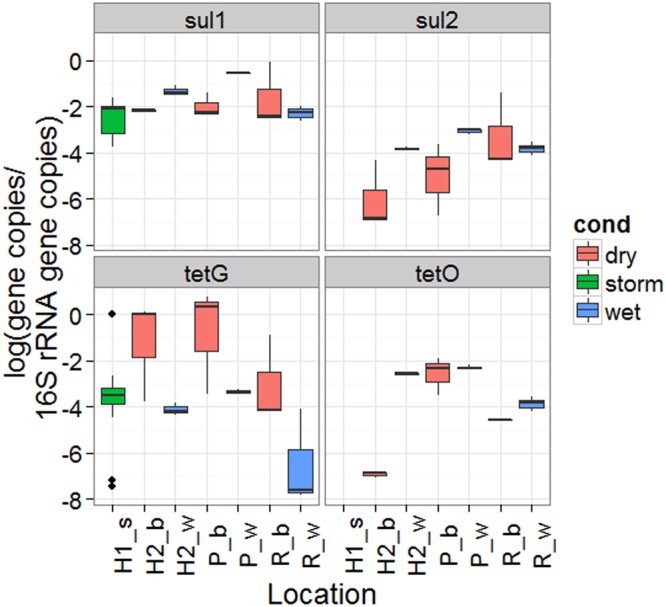
*sul*1, *sul*2, *tet*(G), and *tet*(O) ARG concentrations normalized to 16S rRNA gene copies observed outside of a CSO outfall during three storm events (H1_s), the Hudson at a separate sampling location (H2), the Passaic (P), and Raritan (R) during baseflow (b) and wet weather (w).

Samples collected during baseflow and during or immediately following rainfall events in local waterways were analyzed to provide a broader understanding of ARG during wet weather events in CSO impacted waters. The total (attached plus suspended) 16S rRNA gene copy normalized *sul*1 and *tet*(G) concentrations observed during the three storm events were within the range observed during the baseflow and wet weather sampling in the Passaic River, Hudson River, and Raritan Bay (**Figure 5**). Significantly higher 16S rRNA normalized *sul*1 gene copy concentrations were observed in wet weather in water column from Passaic (*p* = 0.041, both samples collected during low tide) and Hudson Rivers (*p* = 0.013, potentially confounded by tides), but not Raritan Bay (*p* = 1.00). Significant differences were not observed in the water column for *tet*(G), *tet*(O) or *sul*2 between baseflow and wet weather in water or sediment at any site (Supplementary Figure A4).

**FIGURE 6:**
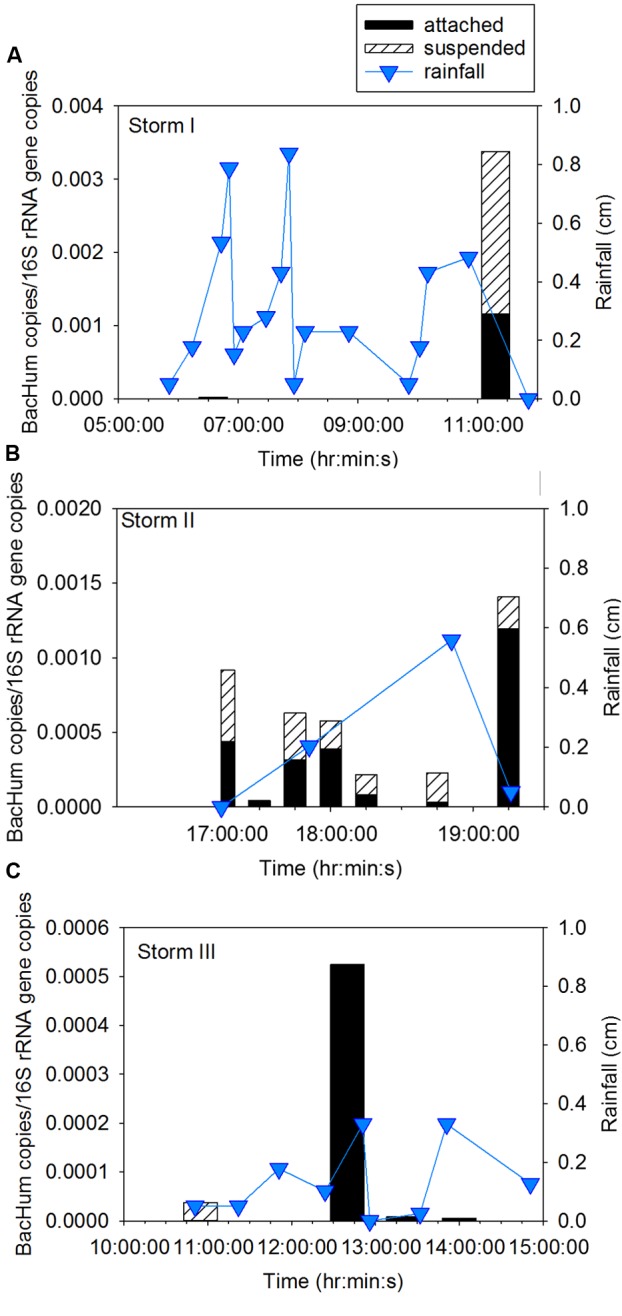
Attached and suspended BacHum gene copies/16S rRNA gene copies and rainfall across **(A)** Storm I, **(B)** Storm II, and **(C)** Storm III. Storm I results represent the average of duplicate samples with a relative percent difference of ±1.7 to 19%.

Attached human fecal indicator marker gene BacHum accounted for 46 ± 8% of total BacHum gene copies in Storm I and 61% ± 29% in Storm II (**Figure 6**). Intra-storm variability of BacHum was evidenced by observation of BacHum in only the suspended fraction early in Storm III then only in the settleable fraction later in the storm. Inter-storm variability in partitioning was observed for 16S rRNA gene copies where the attached portion accounted for 56% ± 0% of total 16S rRNA gene copies for Storm I, 57% ± 21% of total for Storm II, and 70% ± 9% of total for Storm III (Supplementary Figure A5). During Storm II, no differences were observed in attached compared to suspended concentrations of the 16S rRNA gene (*p* = 0.38). Attached 16S rRNA gene copies were greater than suspended 16S rRNA gene copies (*p* = 0.014) for Storm III.

### Timing of Peak Microbial Agent Release

Intra- and inter-storm variability was observed in the concentration of ARG and fecal indicator marker genes. During Storm I, ARG and indicator gene concentrations peaked in the sample collected at the end of the storm. During Storm II, concentrations of *sul*1 and BacHum peaked in the first and final samples, while concentrations of *tet*(G) peaked mid-storm event. For Storm III, *sul*1 concentrations increased across the storm event while the highest BacHum concentrations were observed in the middle of the storm. Inter-storm variability was observed with *sul*1 gene copy numbers significantly lower in Storm III than Storms I and II (both *p* < 0.03). No significant differences were observed between Storms I and II for *sul*1 gene copy numbers (*p* = 1). Differences between *tet*(G) and BacHum copies were not observed between the storms (*p* = 0.2–1.0).

**FIGURE 7:**
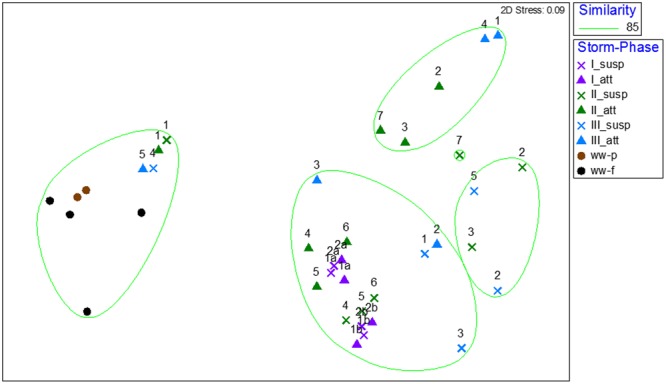
Non-metric multidimensional scaling analysis of bacterial community structures for wastewater influent concentrated by centrifugation (ww-p) or filtering (ww-p), and suspended (x) and attached (triangles) samples collected across three storms (I purple, II green, and III blue). The numbers above the storm sample symbols represent the order of samples (1 being the first sample collected, etc.). The overlay represents clusters with 85% similarity.

Amplicon sequencing was performed to determine when during the storm events CSO effluent samples resembled wastewater and was therefore likely to contain microbial contaminants from wastewater. The bacterial community structure for the attached and suspended fractions observed at the CSO outfall across the storms (**Figures 2C,D, 3C,D** and Supplementary Figures A3B,C) and wastewater concentrated two ways (filter concentrating vs. cell pelleting) were compared using nDMS analysis (**Figure 7**). Four storm samples had 89.4% similarity (Cluster analysis with SIMPROF test *p* = 1 × 10^-3^, Supplementary Figure A6) with the wastewater. Rarefaction curves are included as Supplementary Figure A7. The storm samples with community structures resembling wastewater were marked by increased relative abundances of Bacteroidia (14% ± 2% in wastewater-like storm samples vs. 0.2% ± 0.3% in other storm samples), Clostridia (5.1% ± 0.6% in wastewater-like samples vs. 0.2% ± 0.2% in other storm samples), and Fusobacteria (1.9% ± 1.0% in wastewater-like samples vs. 0.1% ± 0.1% in other storm samples). Both the first attached and suspended samples from Storm II had microbial community structures resembling the wastewater. For Storm III, the final attached and the second to last suspended sample resembled wastewater. Water quality was determined by measuring conductivity and TSS during the storm events. In general, peak conductivity and TSS (**Figures 2, 3** and Supplementary Figure A3) did not occur when CSO effluent microbial communities resembled wastewater.

Of particular interest is the timing of the release of bacterial groups that contain indicator organisms and pathogens. The order Enterobacteriales contains fecal indicators (*Escherichia coli*), several waterborne pathogens (e.g., *E. coli, Salmonella, Klebsiella*, and *Shigella*), and many other bacteria that fit neither of these classifications. By finding the product of the relative abundance of Enterobacteriales and the concentration of 16S rRNA gene copies, one may estimate the concentration of Enterobacteriales in a sample (Supplementary Figure A8). For Storm III, samples with community structures similar to wastewater had 10^1.7^–10^1.8^ greater Enterobacteriales (both > 1 standard deviation above the average Enterobacteriales observed in the other storm samples). This observation was not preserved for Storm II samples with community structures similar to wastewater: the attached and suspended Enterobacteriales were similar to other storm samples (10^7.9^ gene copies in attached sample similar to wastewater vs. 10^7.2^ ± 10^0.4^ gene copies in other attached storm samples; 10^4.2^ gene copies in suspended sample similar to wastewater vs. 10^6.2^ ± 10^1.4^ gene copies in other suspended storm samples).

To better understand the relationship between the attached and suspended microbial communities in CSO effluent the two fractions were compared. The paired attached and suspended microbial communities clustered together without significant differences for 4 out of 14 samples collected across the three storms (**Figure 7** and Supplementary Figure A6). LEfSe analysis revealed 28 discriminating features serve as biomarkers for wastewater compared to the attached and suspended stormwater samples (Supplementary Figure A9). The suspended stormwater community was marked by phyla or members of the phyla Alphaproteobacteria, Actinobacteria, Parcubacteria, and unclassified bacteria. The attached stormwater community was marked by Acidobacteria, Bacteroidetes, Chloroflexi, and three other minor phyla.

## Discussion

### Dynamics of Attached and Suspended ARG Concentrations

Storm samples collected for this study demonstrated that CSO are a source of ARG and that most observed ARG in CSO effluent were attached to settleable particles. Removing the attached fraction of ARG in CSO effluent from this study would result in 0.5-log to 0.9-log removal of *sul*1 and *tet*(G) gene copies. The high percentages of settleable particle associated ARG and fecal indicator marker genes are likely due to ARG and fecal marker genes present in biofilm and cells that preferentially grow attached to particles. When *tet*(G) concentrations were normalized to 16S rRNA gene copies, no significant differences were observed between attached and suspended *sul*1 or *tet*(G) concentrations indicating the higher total bacterial densities in the attached fraction could explain the higher ARG *tet*(G) concentrations observed on the attached phase. In contrast, attached *sul*1 gene copies were greater than suspended concentrations with and without normalization to total bacterial population, indicating that the greater microbial density on the attached phase was not enough to explain why more *sul*1 genes were associated with settleable particles. Here, the greater association of *sul*1 with settleable particles may be due in part to sorption of extracellular DNA, which has been demonstrated to sorb to organic and inorganic clays, soil particles, sand (Lorenz and Wackernagel, 1987), and humic acids (Crecchio and Stotzky, 1998) that would be associated with settleable particles in water.

The percentage of ARG in the attached fractions observed outside a CSO outfall were on the higher end of ranges reported for particle-associated indicator organisms in other studies: 30–60% of total coliform, fecal coliforms, *E. coli* and *Enterococci* partitioned to settleable particles in a stormwater impacted river (Characklis et al., 2005), 40–50% of bacterial indicators were attached to settleable particles in a stormwater impacted stream (Krometis et al., 2007), and 77% of the *E. coli* were attached in CSO effluent (Passerat et al., 2011). When comparing between these studies the differences in methodology for defining the attached and suspended fractions in water samples and for quantifying microbial contaminants should be considered. This study used the methods of Characklis et al. (2005) and Krometis et al. (2007) to separate attached and suspended fractions. Characklis et al. (2005) determined that 80% of suspended particles would remain in the supernatant using this centrifugation procedure. Passerat et al. (2011) used a filtration method (attached > 5 μm; 5 μm > suspended > 0.2 μm) and reported higher fractions of attached indicator organisms than the studies using centrifugation based methods. Another important distinction between this work and previous studies differentiating attached and suspended microbes is that the present study quantified gene copies with qPCR in contrast with the analysis of samples by cultivation based methods. qPCR is generally more sensitive than cultivation based techniques due in part to the fact that it does not discriminate between genes from viable and non-viable sources (i.e., dead cells, extracellular DNA). For BacHum, qPCR can overestimate the concentration of fecal indicators by detecting DNA from non-viable sources and therefore can also overestimate risk. Because transformation of extracellular DNA into competent cells is possible, ARG from non-viable sources detected by qPCR are still potentially of interest for risk assessment.

The timing of peak ARG concentrations is of interest for CSO interventions including end-of-pipe treatment. *sul*1 peak concentrations were observed at the end of Storm I, the beginning and end of Storm II, and at the end of Storm III. While peak *sul*1 concentration was observed early and/or late in the storm events, the timing of peak *tet*(G) concentrations was variable, indicating that treating more than the first and final flush may be desirable. Given that the timing of peak ARG concentrations did not necessarily coincide with the observation of microbial community signatures indicative of wastewater microbial communities, there are likely other sources of ARG in the storm events (e.g., stormwater). The urban area investigated likely has runoff containing feces from domestic and wild animals that could contribute to the indicator organism and ARG loads in CSO effluent. Domestic animals are receiving increasing antibiotic prescriptions and may serve as a reservoir for antibiotic resistant bacteria (Guardabassi et al., 2004). There are also several potential differences between the storms sampled that may contribute to the inter-storm variability in the timing and concentration of ARG in CSO effluent including differences in rainfall intensity, timing of the storm with respect to diurnal flow, and, in particular, in the amount of rainfall preceding the targeted storm, as discussed further in section “Timing and Partitioning of Wastewater Signatures and Fecal Indicators in CSO Effluent.” Sampling of more storm events could reveal common patterns not observed here given that only three storm events were captured.

Sampling for total ARG performed during baseflow and wet weather conditions in the Passaic River, Hudson River, and Raritan Bay provided local comparison for the three storm samplings. The total ARG (attached plus suspended) concentrations observed in the storm samples were within the range observed during baseflow and wet weather conditions in local water ways. During wet weather ARG concentrations in surface water would be lowered by dilution, an observation noted during historic flooding in Colorado (Garner et al., 2016), if rain and stormwater have lower ARG. However, wet weather introduces sources including CSO effluent, stormwater, and resuspension of settled ARG. These phenomena may explain the variability in the concentrations of ARG observed in the river and bay samples during wet weather, all of which are impacted by CSO, compared to the end-of-pipe ARG observed in the storm samples. Compared to the maximum concentrations of ARGs observed in wastewater influent (data not shown), ARG concentrations observed at the CSO outfall represent <1–40% of the *sul*1 and <1–5% of the *tet*(G) observed in wastewater influent. Dilution of ARG may explain why the lowest *sul*1 concentrations were observed in Storm III, which had the greatest cumulative rainfall. Concentrations of wastewater micropollutants such as hormones were found to decrease with increasing flow because of such dilution (Phillips et al., 2012).

### Timing and Partitioning of Wastewater Signatures and Fecal Indicators in CSO Effluent

Microbial community structures with high similarity to wastewater were observed at select time points early or late in the storm event outside of the CSO outfall during wet weather. This observation is consistent with other studies that reported peak concentrations of indicator organisms of fecal pollution early and late in the hydrograph for CSO effluent, CSO-impacted surface waters (Puerta et al., 2002; Passerat et al., 2011), and stormwater runoff (Puerta et al., 2002; Krometis et al., 2007). During CSO this first flush phenomena has been attributed to the increased shear stress sewer solids experience at the higher velocities occurring during wet weather events, which scour stationary bed solids that accumulated in combined sewers pipes during dry periods (Hvitved-Jacobsen et al., 2013). Sewer solids have been linked to the indicator organism loads in CSO effluent (Passerat et al., 2011). For Storm II, the microbial community signature of wastewater was observed at the same time for attached and suspended samples. Given that precipitation occurred earlier in the day before this storm (Supplementary Figure A1) the coincidence of the release of attached and suspended microbes resembling wastewater may be because settleable particles were recently deposited and therefore loose. A microbial community structure with a wastewater signature was also observed in the fourth suspended and fifth (final) attached sample from Storm III. The timing difference may be due to velocities below the critical velocity during the lull in rainfall, allowing for flushing of suspended bacteria and for settling during lower flow followed by resuspension of solids when the rainfall resumed. More robust sampling of wastewater influent and sampling of sewer solids may have indicated a wastewater association with more samples and is recommended for future studies along with collecting stormwater samples.

The timing of peak BacHum observations did not coincide with the microbial signatures of wastewater. Partitioning of BacHum to settleable particles was variable across the storms. BacHum is a highly sensitive but moderately specific human fecal indicator gene exhibiting some cross amplification with feces from deer, dogs, geese, and gulls (Ahmed et al., 2009; Boehm et al., 2013), which can be expected to be present in stormwater. Thus, the BacHum peak concentrations observed mid-storm may be representative of such cross amplification. The attached and suspended Enterobacteriales were determined given that this group includes indicator organisms such as *E. coli* and select waterborne pathogens such as *Salmonella* and *Shigella*. Enterobacteriales concentrations were relatively consistent across the storms except for during Storm III where their concentrations peaked at times when the wastewater signatures were observed. The weak correspondence with wastewater signatures is likely because there are many commensal organisms in the Enterobacteriales order. Given the limitations of the short reads generated by amplicon sequencing, sequences were not identified below this taxonomic level.

Understanding the differences between the attached and suspended microbial communities is of interest for understanding potential removal of groups containing pathogens and for understanding the impact on surface water microbial ecology. The attached and suspended microbial communities observed in CSO effluent clustered together without significant differences for 4 out of 14 samples collected across the three storms (**Figure 7** and Supplementary Figure A6). LEfSe analysis revealed 28 discriminating features serve as biomarkers for wastewater and CSO resembling wastewater compared to the attached and suspended stormwater microbial communities (Supplementary Figure A9). The ability to identify biomarkers of attached compared to suspended stormwater microbial communities reinforces observations that the fraction attached to particles varies for different microbes (Suter et al., 2011). LEfSe analysis indicated that the phyla Firmicutes and Epsilonproteobacteria, which includes the order Enterobacteriales, were biomarkers of wastewater. Other researchers also observed genera belonging to Epsilonproteobacteria to be sewage biomarkers (McLellan et al., 2015). CSO events were also associated with increases in Epsilonproteobacteria and Clostridia, as well as Gammaproteobacteria, Bacteriodia, and Bacteroidetes in the receiving water (Newton et al., 2013). Differences in the sewage signatures observed between these studies are likely due to a combination of differences in targeted variable region known to impact amplicon sequencing results (Tremblay et al., 2015) and geographical and population differences which can alter the sewage microbiome (Newton et al., 2015). The storm samples that did not have wastewater signatures had higher relative abundances of Alphaproteobacteria and Actinobacteria compared to the storm samples clustering with wastewater. Other researchers looking for biomarkers of stormwater also observed elevated relative abundances of genera in the class Actinobacteria along with Gammaproteobacteria and Betaproteobacteria (McLellan et al., 2015), with the same caveats as above on targeted variable region.

### Implications for Design and Operation of Hydrodynamic Separators

Treatment by hydrodynamic separation removes settleable materials by gravity and extension of the particle flow path (Alkhaddar et al., 2001). Therefore, one cannot directly compare the centrifugal forces used in this study to separate settleable particles to the centrifugal forces in hydrodynamic separators, because that is a minor mechanism for particle removal. However, one can optimize these systems for removal of the settleable particles. Wilson et al. (2009) determined that Peclet number (a function of settling velocity, hydrodynamic separator dimensions, and flow rate) could be used to predict removal efficiency for hydrodynamic separator treatment devices. Particle settling velocity was determined using previously reported characteristics of the attached and suspended fractions separated by the centrifugation technique applied here (Krometis et al., 2007) (Supplementary Table A1). As an example, using these settling velocities and example flow and dimensions provided by Wilson et al. (2009) for a select unit, one would estimate 0.3–35% removal of suspended particles. However, units can be sized to improve these removal efficiencies. Given that peak concentrations of ARG and fecal indicators were observed at various times during the storm events, removing settleable particles throughout the storm rather than targeting first or final flush may be needed. Even with 100% removal of ARG on settleable particles, further treatment may be desirable, particularly if one assumes the ARG present in the untreated wastewater fraction of CSO effluent are in viable cells. Often disinfection is included post-separation [e.g., (Patoczka et al., 2016)]. Disinfection has been shown to inactivate microbes carrying ARG (Dodd, 2012) and UV disinfection can help destroy ARG (McKinney and Pruden, 2012). Thus, the relative concentration (compared to baseflow, which also showed variability) and compartment (viable vs. non-viable) may be of consideration for determining treatment goals.

## Conclusion

This study highlights the presence, partitioning, and intra- and inter-storm variability of ARG, indicator marker genes, and microbial communities at a CSO outfall during wet weather. CSO were found to be a source of ARG. Generally, ARG were detected in higher concentrations in the attached compared to the suspended fraction of CSO effluent while BacHum was more variably associated with the different fractions. Higher total bacterial concentrations on the settleable particles could explain the difference between attached and suspended *tet*(G) concentrations. A microbial community structure with a wastewater signature was observed at select time points at the beginning or end of the storm but did not necessarily coincide with peak ARG and BacHum concentrations indicating that treatment of more than the first and final flush may be desirable. Results of this study indicate further treatment would be needed after separation of settleable particles for ARG given that ARG would remain in the suspended fraction of water during CSO events.

## Author Contributions

Conception, design, data analysis, and interpretation by AE, NF, and HDR. Data collection performed by AE and HDR. Article drafted by AE and NF.

## Conflict of Interest Statement

The authors declare that the research was conducted in the absence of any commercial or financial relationships that could be construed as a potential conflict of interest.
