# Supplementary material for: Partitioning of Antibiotic Resistance Genes and Fecal Indicators Varies Intra and Inter-Storm during Combined Sewer Overflows
Source: Front Microbiol. 2017 Oct 20;8:2024. doi: 10.3389/fmicb.2017.02024 (PMC5655003; doi:10.3389/fmicb.2017.02024)
Supplement: Supplementary file 1 [file Data_Sheet_1.pdf]

Partitioning of antibiotic resistance genes and fecal indicators varies intra and inter-storm during combined sewer overflows

Alessia Eramo[a], Hannah Delos Reyes[a], N.L. Fahrenfeld[a]*

[a]Department of Civil and Environmental Engineering, Rutgers, The State University of New Jersey, 96 Frelinghuysen Rd., Piscataway, New Jersey 08854, United States

Correspondence: nfahrenf@soe.rutgers.edu, (848)-445-8416.

Supplementary Material

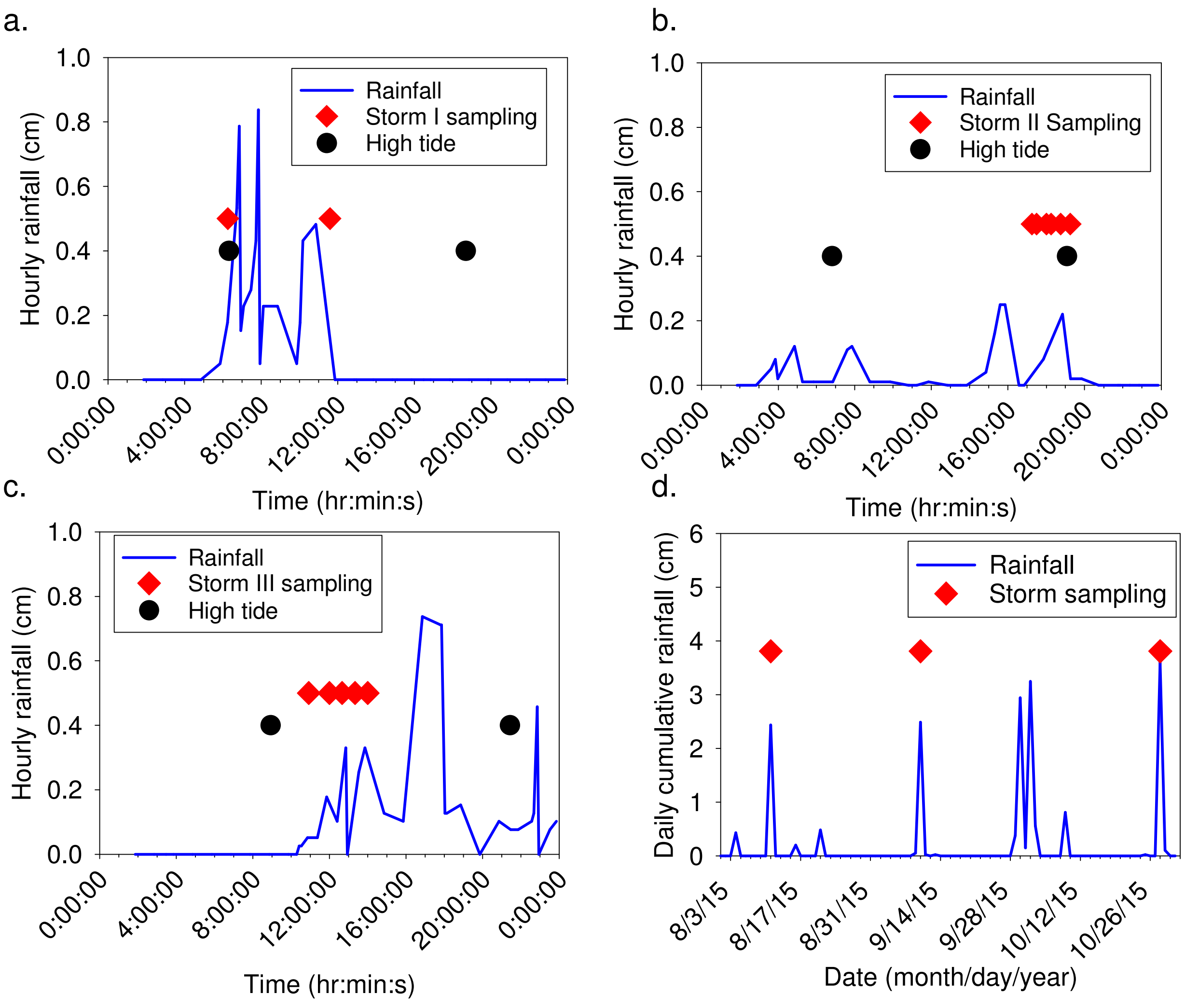

Fig. A1 Hourly precipitation, sampling time, and time of high tide for the day of (a) Storm I, (b), Storm II, and (c) Storm III. (d) Daily cumulative precipitation across the study period. Precipitation data presented for Newark, NJ, the location of the nearest rain gauge.

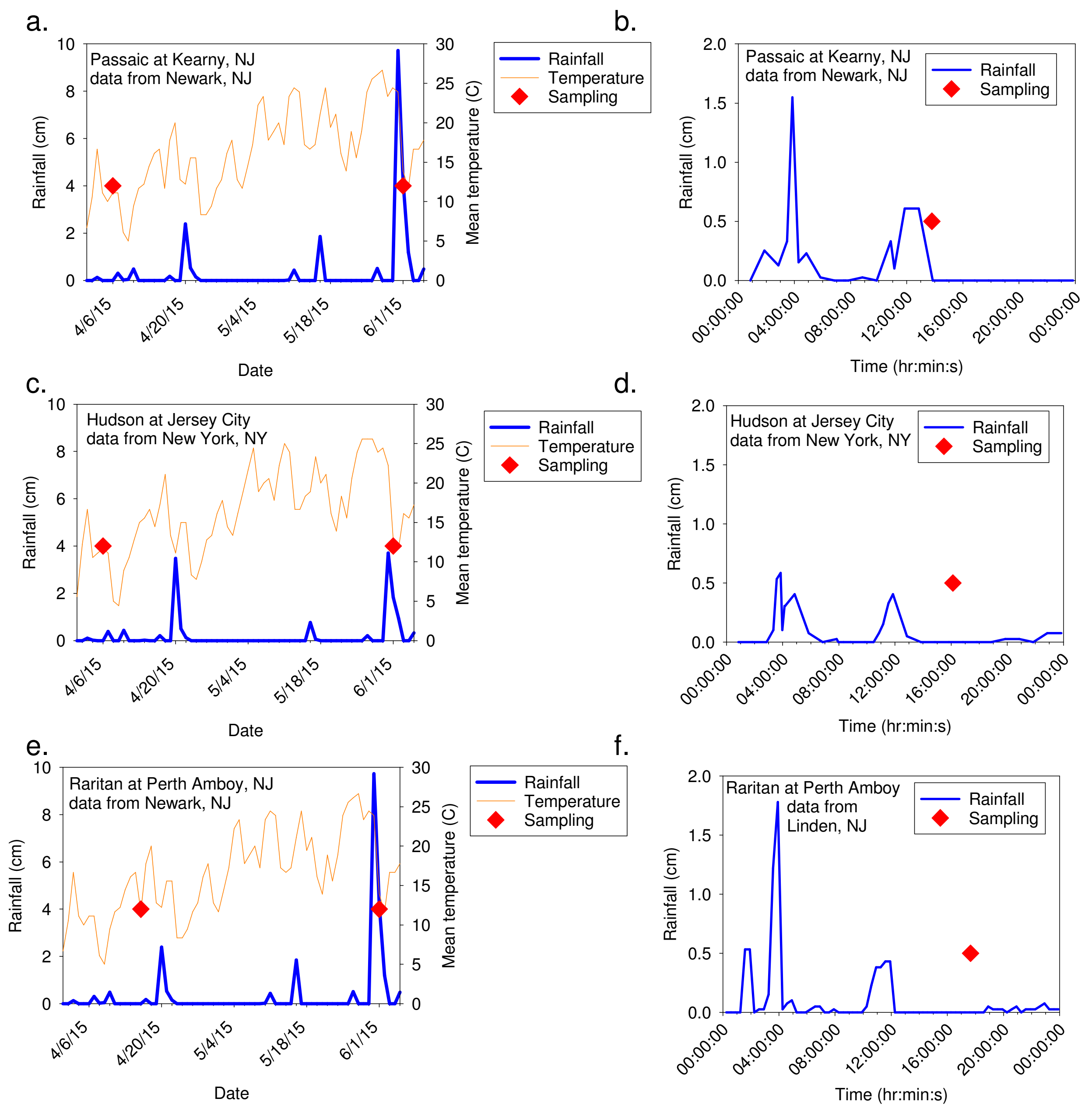

Fig. A2 Precipitation and mean daily air temperature data for nearest gauge for the (a) daily for the Passaic (data from Newark, NJ), (b) hourly for Passaic during wet weather event (data from Newark, NJ), (c) daily for the Hudson at Jersey City (rainfall data from New York, NY), (d) hourly for the Hudson during wet weather event (data from New York, NY), (e) daily for Raritan Bay (data from Newark, NJ), and (f) hourly for wet weather event (data from Linden, NJ).

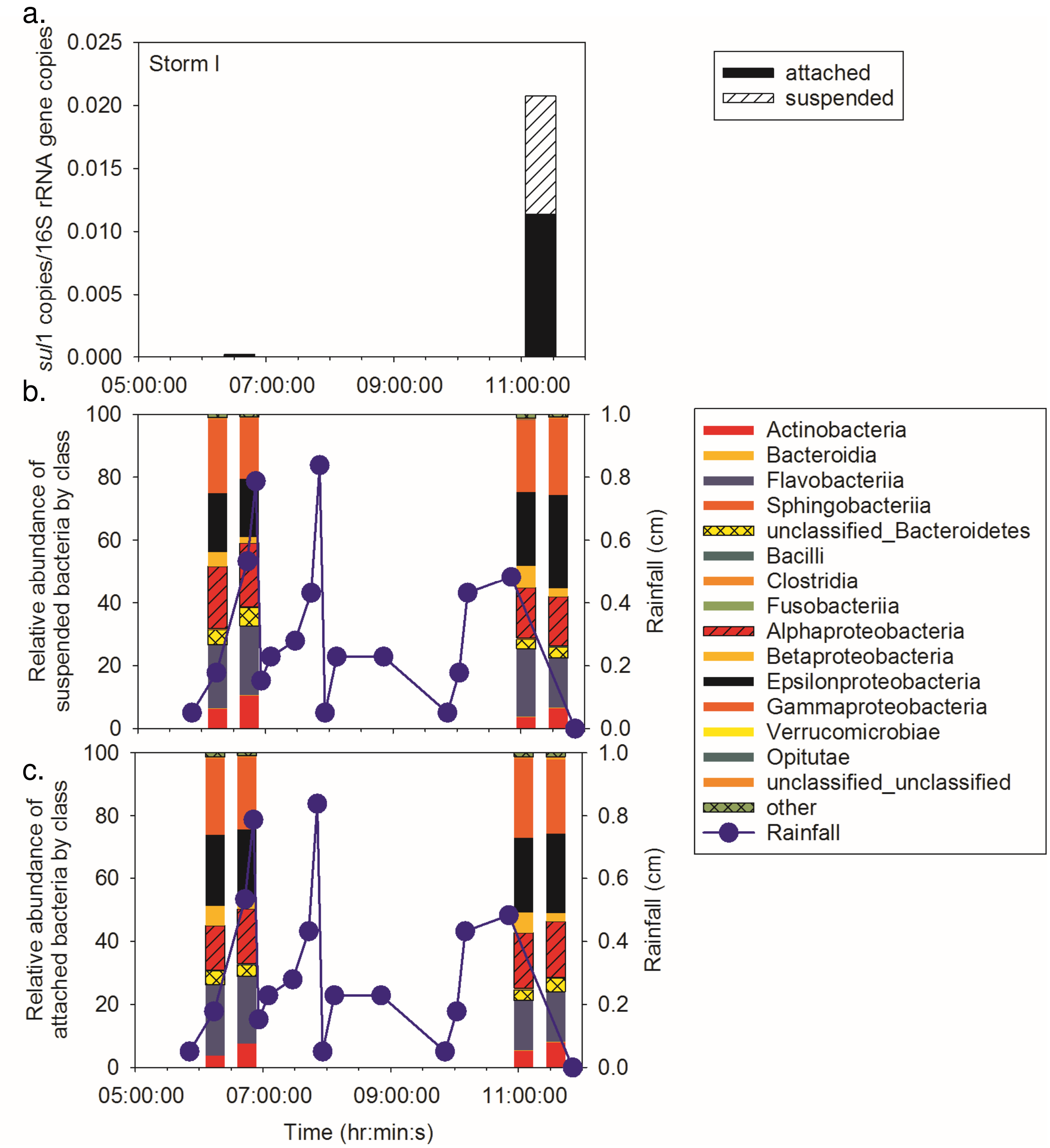

Fig. A3 Storm I a. ARG flux, b. suspended bacteria by class and rainfall, c. attached bacteria by class and rainfall. Sequencing results shown are for replicate samples collected at the same time. ARG results represent the average of duplicate samples with a relative percent difference of ±0.3-2.4%.

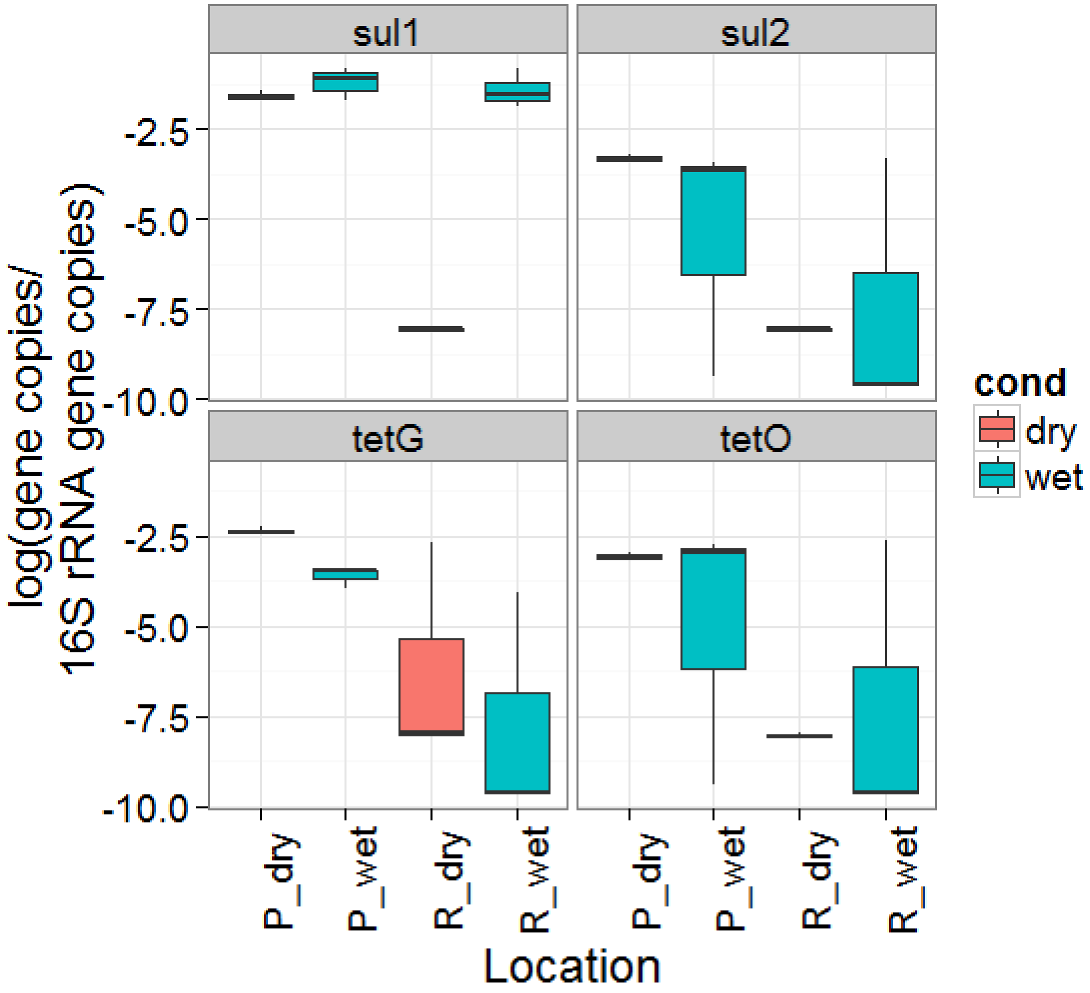

Fig. A4 Concentrations of ARG normalized to 16S rRNA from composited sediment samples collected during base flow (dry) or wet weather conditions in the Passaic River (P) and Raritan Bay (R).

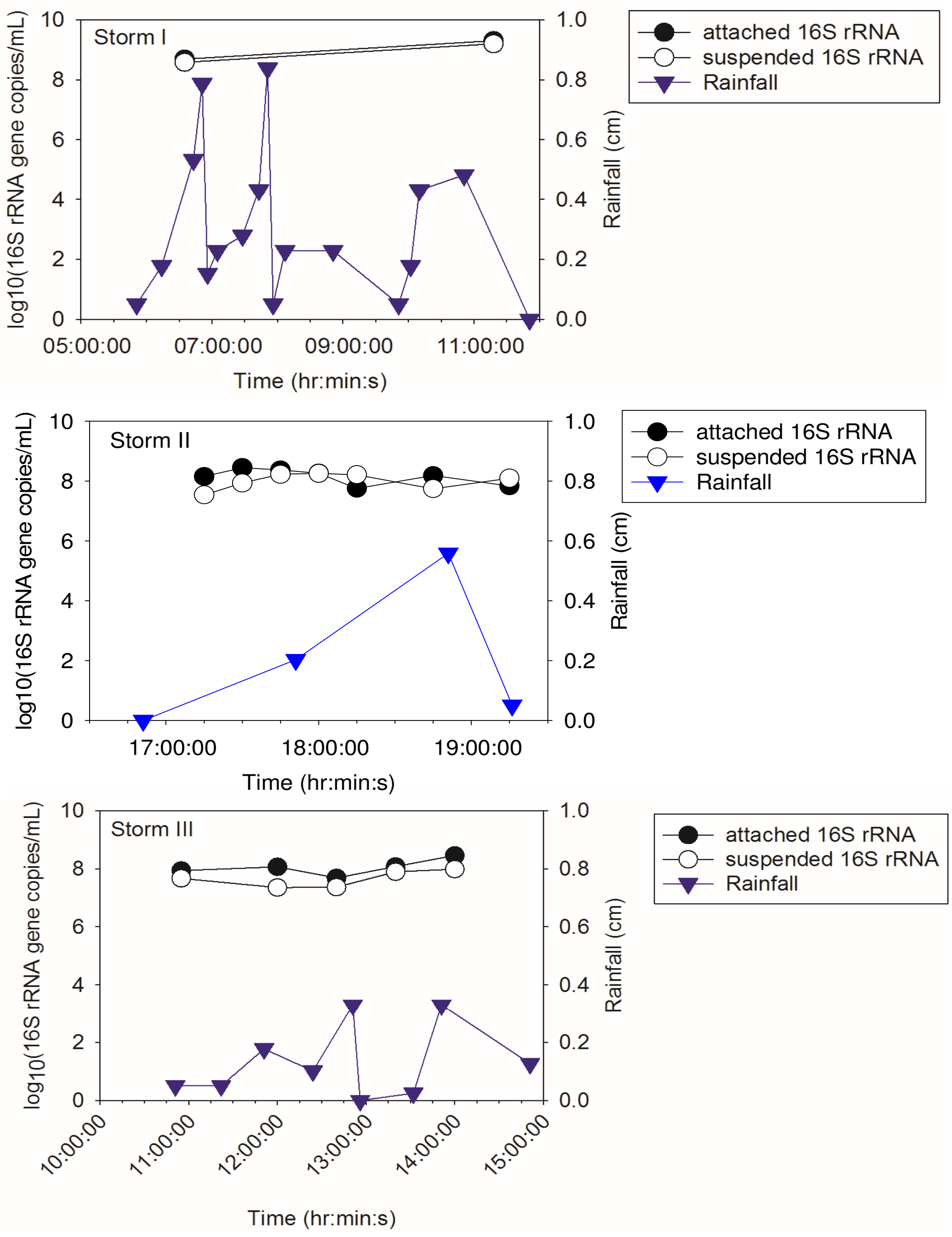

Fig. A5 Attached and suspended 16S rRNA gene copies/mL and rainfall across (a) Storm I, (b) Storm II, and (c) Storm III.

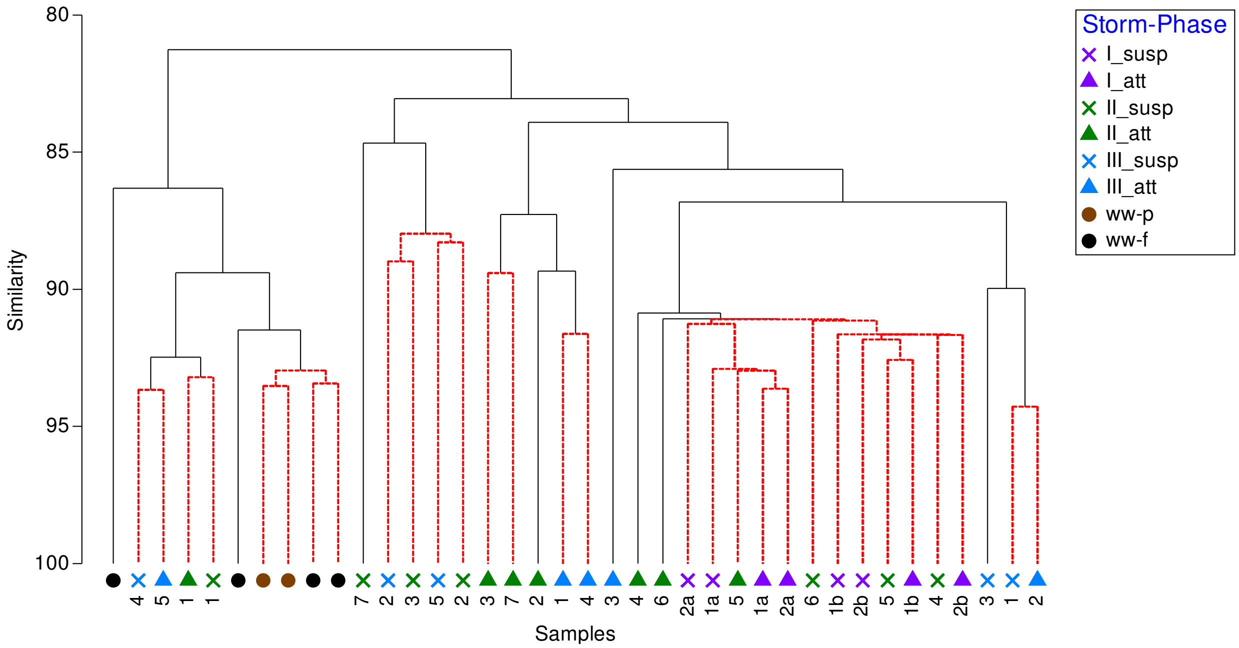

Fig. A6 Cluster analysis for bacterial communities at the class level. Numbers refer to sample number (1 for first, etc.) for a given storm, "a" and "b" refer to replicates. Red branches connect samples with bacterial community structures that were not different via SIMPROF test.

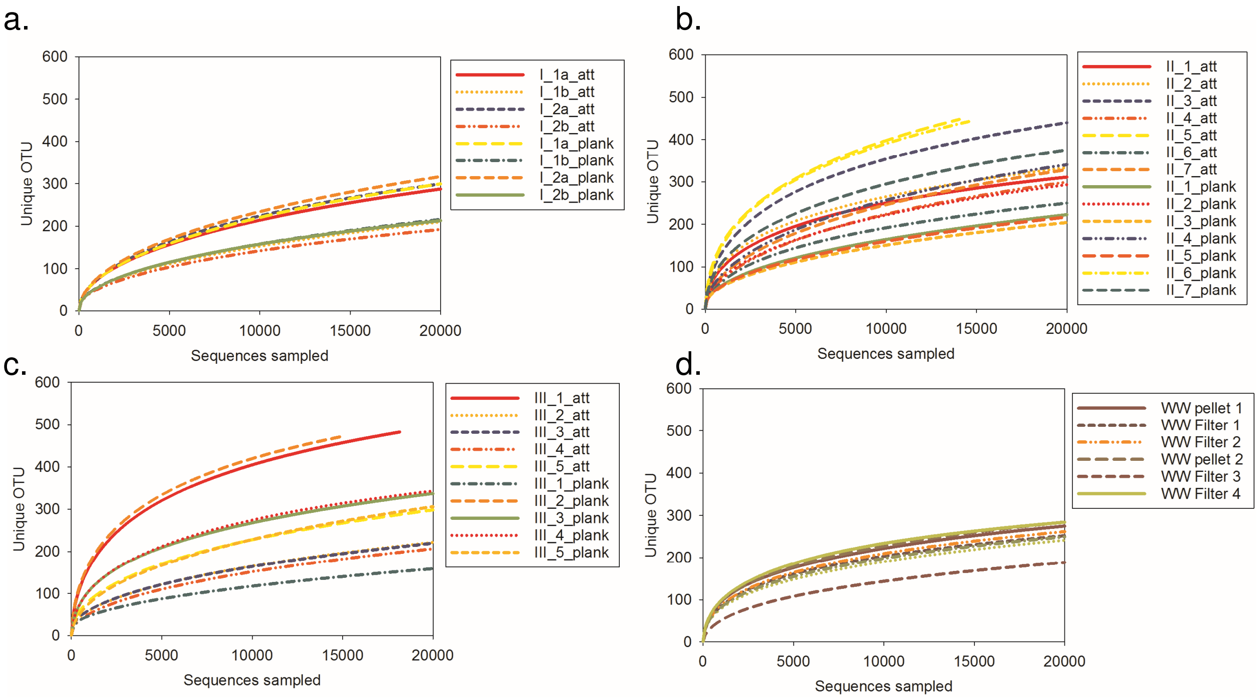

Fig. A7 Rarefaction curves for (a) Storm I, (b) Storm II, (c) Storm III, and (d) simulated CSO. Numbers for storm samples indicate sample order. Attached samples are indicated by "att" and suspended samples are indicated by "plank." Storm replicates are labeled as "a" and "b." CSO samples were diluted wastewater (WW) concentrated by filtering or by centrifugation ("pellet").

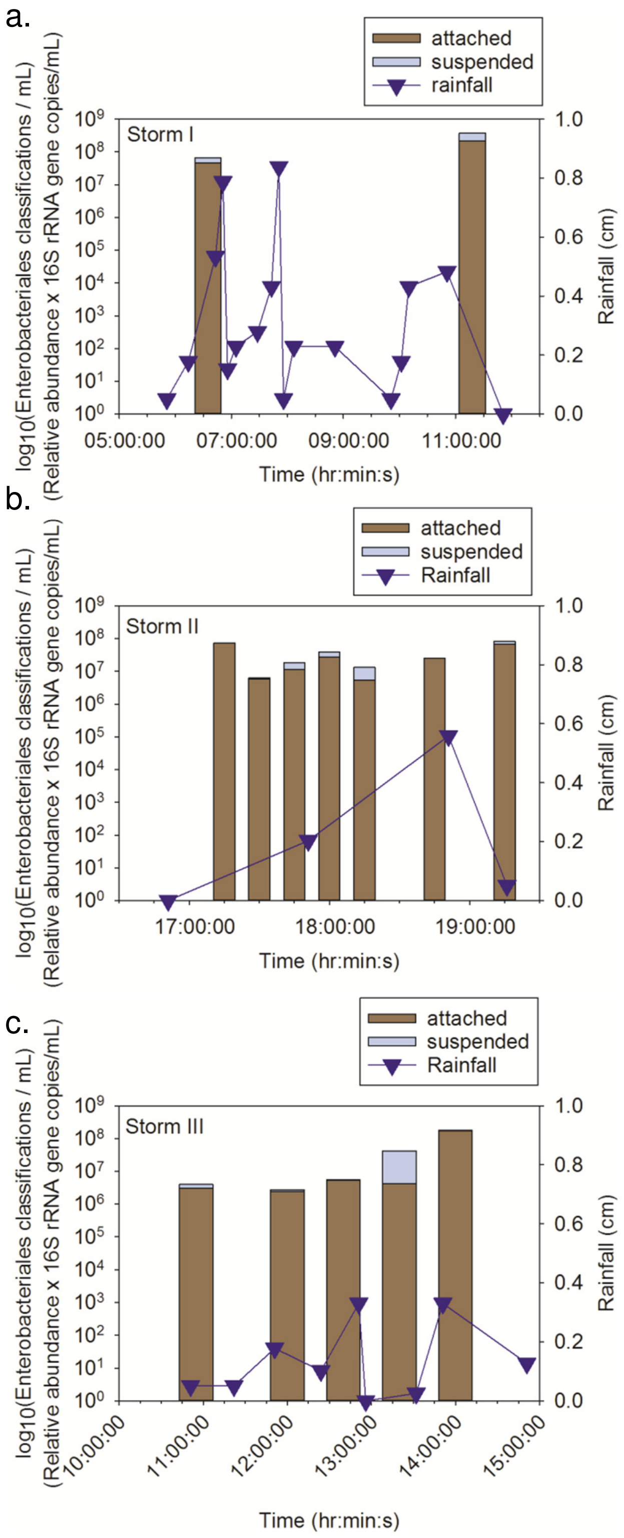

Fig. A8 Attached and suspended calculated *Enterobacteriales* and rainfall across (a) Storm I, (b) Storm II, and (c) Storm III and rainfall.

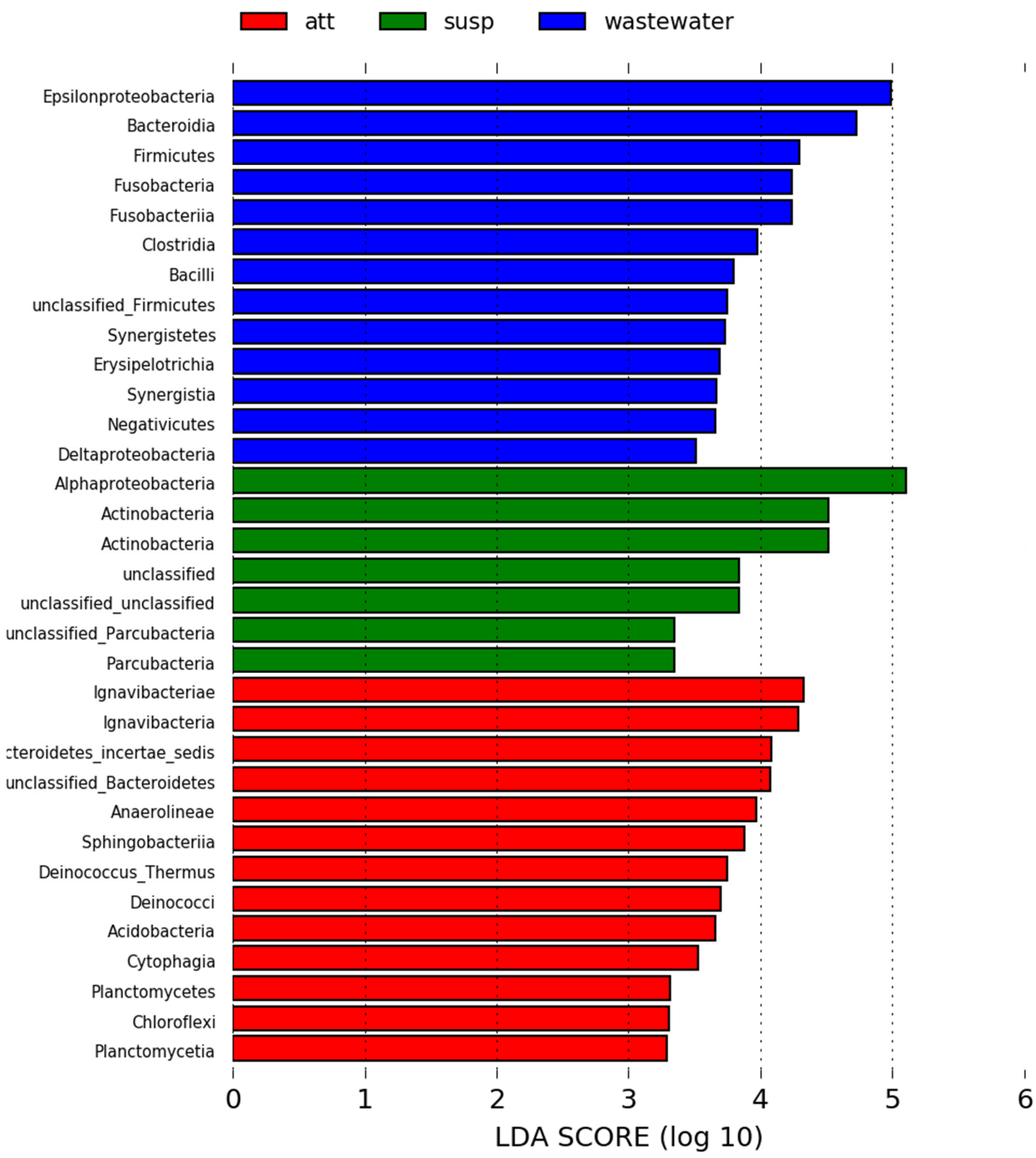

Fig. A9 LEfSe analysis on wastewater, attached storm, and suspended storm (excluding storm samples clustering with wastewater) indicates biomarkers for each matrix at the Phylum and Class level.

Table A1. Example removal efficiency for minimum and maximum surrogate particles representing the attached and suspended fractions using an example hydrodynamic separator unit (i.e., Stromceptor STC 4800 with 3.7 m diameter, 3.4 m settling depth, and flow rate 0.051 m^3/s). Hydrodynamic separators can sized during design to improve removal. For these estimates the following constants were used: kinematic viscosity water $\nu = 1.12\times10^{-6}$ m^2/s, gravity 9.81 m/s^2, and water density $\rho = 999$ kg/m^3.

Variables (units)	Values				Reference
Fraction	attached, minimum	attached, maximum	suspended, minimum	suspended, maximum	(Krometis et al., 2007)
Diameter, D (μM)	5	60	5	40	
Particle density, ρ_s (kg/m^3)	2.65×10^3	2.65×10^3	1.05×10^3	1.05×10^3	
Settling velocity, V_s (m/s)	1.50×10^{-5}	2.09×10^{-3}	4.65×10^{-7}	2.97×10^{-5}	(Cheng, 1997)
Peclet number, P (unitless)	3.7×10^{-3}	5.2×10^{-1}	1.1×10^{-4}	7.3×10^{-3}	Eq. 3 in (Wilson et al., 2009)
Removal efficiency, η (%)	0.26	35	0.01	0.51	Eq. 7 in (Wilson et al., 2009)

References

Cheng, N.S. (1997). Simplified Settling Velocity Formula for Sediment Particle. *Journal of Hydraulic Engineering* 123(2). doi: doi:10.1061/(ASCE)0733-9429(1997)123:2(149).

Krometis, L.-A.H., Characklis, G.W., Simmons Iii, O.D., Dilts, M.J., Likirdopulos, C.A., and Sobsey, M.D. (2007). Intra-storm variability in microbial partitioning and microbial loading rates. *Water Research* 41(2)**,** 506-516. doi: 10.1016/j.watres.2006.09.029.

Wilson, M.A., Mohseni, O., Gulliver, J.S., Hozalski, R.M., and Stefan, H.G. (2009). Assessment of Hydrodynamic Separators for Storm-Water Treatment. *Journal of Hydraulic Engineering* 135(5). doi: doi:10.1061/(ASCE)HY.1943-7900.0000023.
